# Heated indoor swimming pools, infants, and the pathogenesis of adolescent idiopathic scoliosis: a neurogenic hypothesis

**DOI:** 10.1186/1476-069X-10-86

**Published:** 2011-10-05

**Authors:** Marianne E McMaster

**Affiliations:** 1 1Scottish National Paediatric Spine Deformity Centre, Royal Hospital for Sick Children, Edinburgh, UK

## Abstract

**Background:**

In a case-control study a statistically significant association was recorded between the introduction of infants to heated indoor swimming pools and the development of adolescent idiopathic scoliosis (AIS). In this paper, a neurogenic hypothesis is formulated to explain how toxins produced by chlorine in such pools may act deleteriously on the infant's immature central nervous system, comprising brain and spinal cord, to produce the deformity of AIS.

**Presentation of the hypothesis:**

Through vulnerability of the developing central nervous system to circulating toxins, and because of delayed epigenetic effects, the trunk deformity of AIS does not become evident until adolescence. In mature healthy swimmers using such pools, the circulating neurotoxins detected are chloroform, bromodichloromethane, dibromochloromethane, and bromoform. Cyanogen chloride and dichloroacetonitrile have also been detected.

**Testing the hypothesis:**

In infants, the putative portals of entry to the blood could be dermal, oral, or respiratory; and entry of such circulating small molecules to the brain are via the blood-brain barrier, blood-cerebrospinal fluid barrier, and circumventricular organs. Barrier mechanisms of the developing brain differ from those of adult brain and have been linked to brain development. During the first 6 months of life cerebrospinal fluid contains higher concentrations of specific proteins relative to plasma, attributed to mechanisms continued from fetal brain development rather than immaturity.

**Implications of the hypothesis:**

The hypothesis can be tested. If confirmed, there is potential to prevent some children from developing AIS.

## Background

### Epidemiological studies

Findings from two previous studies showed a statistically significant association of adolescent idiopathic scoliosis (AIS) in patients and vertical spinous process asymmetry in controls, each group of which used heated indoor swimming pools in the first 12 months of life [1,2]. Patients and controls were matched for age, sex, ethnic origin, and social status, and did not have previous health problems. The odds of being diagnosed with AIS in later life was about three times higher in children introduced to a pool within their first year than in those who were not. 61% of controls in the first study [1] and 83% of controls in the second study [2] had spinous process asymmetry with the child standing upright. The forward bending test to assess the controls [3-5] was not used because of its low predictive value. All patients and controls were full term at birth.

### Scoliosis

**Figure 1:**
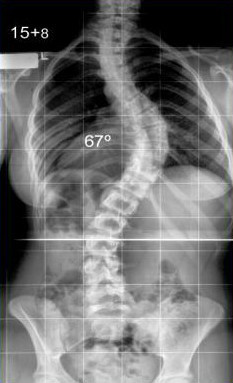
**A 15 year old girl with a right thoracic adolescent idiopathic scoliosis**.

(Figure 1). Scoliosis is diagnosed when the natural perpendicular spine of a teenager, when viewed from the back, develops an S-shape. Viewed from the side the normal thoracic kyphosis, an anteroposterior curve beginning at the base of the neck and extending to the waist, appears flattened. The rib cage becomes distorted because of the three dimensionality of the deformity. When no cause for the scoliosis is identified, the term idiopathic is used. Hence, for diagnosis in teenagers at and after their 10th birthday, the acronym AIS is applied. There are no symptoms before the onset of AIS and there are no clinical, radiological, or biological markers to predict the onset of the condition in pre-pubescent children. Most patients with AIS are female. Children with early-onset scoliosis (infantile and juvenile idiopathic scoliosis) are not included in this study. The role of genetic factors in the development of idiopathic scoliosis is widely accepted, but no causative genes of AIS have been identified [6]. The complexity of causation is shown by the fact that present treatment for AIS is based on mechanical interpretation of pathogenesis, without any biological understanding of the cause. There are likely to be several factors contributing to the etiopathogenesis, and these factors are not necessarily the same in all patients--ie, AIS is a final common pathway deformity. Furthermore, the spectrum of disorder ranges in severity from minimal to very severe, and whether abnormalities thought to be linked to causation are cause or effect is difficult to establish. Finally, the difficulty that geneticists are having with this complex deformity should be acknowledged.

### Formulation of novel neurogenic hypothesis for AIS

The purpose of this paper is to formulate a novel hypothesis to explain how the epidemiological findings might be related to the expression of idiopathic scoliosis developing in adolescents. This paper investigates the possibility that neurotoxic lipid-soluble, volatile, chlorinated gases such as trihalomethanes (THMs) and cyanogens, generated by chlorination of the pool water, are absorbed by the infant and access the infant's central nervous system (CNS) to initiate AIS later in development. This neurogenic hypothesis is not claimed to be the underlying factor of all AIS. A preliminary report of the hypothesis has been presented [7].

### Presentation of the hypothesis

#### Neurotoxic substances produced in swimming pools

In most countries there are guidelines only for the maintenance of public pools. Because bacteria in the water change with the range of bathers, maintenance of the correct concentration of disinfection is difficult. Chlorination of heated indoor swimming pools is crucial to prevent serious health problems from viruses, bacteria, fungi, and protozoa. Chlorination produces THMs that are neurotoxic. Heated chlorinated pool water also generates neurotoxic lipid-soluble, volatile, chlorinated gases.

Aggazzotti [8] undertook blood and breath analyses as biological indicators of exposure to THMs to assess the uptake in five competitive swimmers (three men and two women) with a mean age of 18·6 years (range 17-21). One sample was obtained every month from each swimmer. After resting in the pool area followed by training in the water, samples from bathers were medically assessed. Analyses were done by gas chromatography to detect THMs. Bromodichloromethane, dibromochloromethane, bromoform, and chloroform were detected. In another study, cyanogen chloride and dichloracetonitrile [9] were detected. All are listed as hazardous substances [10].

Cyanogen chloride has an upper 15 min exposure level of 0·77 mg/m^3 ^in air. [11-13]. Chloroform has a long-term 8 h exposure limit of 9·8 mg/m^3 ^[14-16]. Henry's law partial pressure for chloroform is 185 atm, and for cyanogens chloride is 108 atm. Although it is difficult to relate a 15-min maximum exposure, cyanogen chloride has at least a log 2 to log 3 higher toxicity than does chloroform. The concentrations of chloroform and cyanogens chloride in the atmosphere of heated indoor swimming pools are unknown. However, if there is no agitation of the water then concentrations can rise in solution, but activity such as swimming and splashing will release the gases.

### Entry of neurotoxic substances to infant's body

Entry of any of the above mentioned neurotoxic substances to an infant's body could be oral, dermal, though inhalation, or a combination of these routes. Infants have a larger surface area of skin and adipose tissue relative to weight than do adults, allowing more cutaneous permeability into an immature CNS. In babies, inhaled doses might be greater than those in adults since the rapidly developing respiratory system has much less surface area [17]. On average, the increase in respiratory tract exposure (per unit surface area) could be twice as great in babies as in adults [18]. Lung alveolar surface area for the size of the infant is greater than that of adults, which, together with children's higher ventilation rates, contributes to children's increased absorption through inhalation. Therefore pulmonary ventilation, due to physical stress such as swimming, will substantially increase any neurotoxin uptake by the infant.

### Entry of neurotoxic substances to infant's CNS

The fastest brain growth occurs in the last few weeks of fetal life and the first few months after birth in the full-term infants. Growth of the many brain structures occurs in two stages: first the developmental process and then maturity. The intricate sequence of many anatomical and neurochemical growth spurts in each region of the brain is likely to have its own slightly different period of heightened vulnerability [19].

Passage of such neurotoxic substances from blood to CNS can occur through several routes--namely, the blood-brain barrier (BBB), which is used as a convenient short-hand to describe the whole range of mechanisms that control the internal environment of the brain [20]; the blood-cerebrospinal fluid barrier (B-CSF-B); and the barrier-deficient circumventricular organs (CVOs) [21]. Saunders and colleagues [22] state that dysfunctional brain-barrier mechanisms contribute to the pathology of neurological conditions. The choroid plexuses, mainly involved in the production of CSF, are involved in a variety of neurological disorders [23]. There are eight CVOs but only the pineal gland has been extensively researched with regard to AIS [24]. CVOs regulate the autonomic nervous system and act as transducer signals between blood, other centres of the brain, and CSF [25]. The choroid plexuses are considered by some to be CVOs [21].

Adinolfi [26] believed that the BBB was immature in infants until the age of 6 months. However, Saunders and colleagues [27] established that intercellular tight junctions between cerebral endothelial cells in the BBB are well developed from early embryonic life, and they refute the earlier notion of the BBB being immature at birth. Nonetheless, because of the low molecular weight of the detected THMs, these toxins would be capable of entering the developing brain, especially early on, and Saunders states that recent evidence suggests that this may be predominantly via the CSF [28]. The pathway for compounds of low molecular weight, which is much more permeable in the developing choroid plexuses, also seems to be transcellular rather than paracellular via tight junctions [29]. During the first 6 months of life, CSF contains higher concentrations of proteins that are immunologically identical to proteins in plasma; this effect is attributed to mechanisms continued from fetal brain development rather than immaturity [30]. The clinical significance of this vulnerability might be that a large proportion of toxins that bind to plasma proteins cross the choroid plexus [31] and diffuse into the CSF, from where they can reach the individual brain structures. Fry and colleagues state that lipid-soluble molecules are able to diffuse across the cell membrane of cerebral capillaries and into the CNS with relative ease [25]. Because of the infant's proportionally high volume of adipose tissue, this could be an indirect route to the CNS.

### CNS vulnerability to neurotoxic assaults

Assessment of the potential of individual environmental chemicals to produce damage in an infant is crucial, since very few chemicals are tested for developmental neurotoxicity during the risk assessment process [32].

Key factors are lipid solubility, protein binding (if any), and whether these compounds are substrates for efflux transporters such as P-glycoprotein (Prof N Saunders; personal communication). These molecules are so small that they could enter the brain, irrespective of any barrier mechanisms, and they do not have the right chemical groups to be substrates for P-gylcoprotein. The neurogenic hypothesis suggests vulnerability of the developing brain to neurotoxins.

Studies assessing microanatomical development of the brain show that many brain structures have differing peak periods of growth. Therefore, toxic exposures at particular times would differentially affect the structures undergoing peak development. These periods of vulnerability need consideration in assessment of any neurotoxic effects on the developing CNS.

### Time lag

Per Eriksson [33] refers to the perinatal development of the brain and the so-called brain growth spurt, which begins during the third trimester of pregnancy and continues throughout the first 2 years of life. In addressing the vulnerability of the infant, lipid-soluble chemicals can cause damage, even very minor damage, to a brain structure at an early developmental stage, which will be amplified as the organism grows, undergoes physiological change, or comes under stress. Nevertheless, the time lag in the manifestation of a scoliosis at the onset of adolescence needs explanation. Because AIS is associated with puberty, I hypothesise that whatever the putative effects of the neurotoxic products on the brain, the process of puberty with its increased growth velocity has a role in its phenotypic expression. The neurogenic hypothesis for AIS outlined here accords with the concept of developmental origins of health and disease reviewed by Gluckman and Hanson [34], implying delayed epigenetic effects of neurotoxic substances. Studies of the toxicity of xenobiotic compounds in children have shown the potential for either acute or chronic exposure to result in serious malfunctions at a later age [35]. The long-term consequences of an early neurotoxic assault on the developing brain are recognised--for example, bisphenol A, which is known as an oestrogenic chemical that has been banned in Canada from such products as baby bottles [36] because of its effects on prostate and brain development. Phthalate esters, another plasticiser used in the manufacture of toys, are known to be antiandrogenic, and exposure has been shown to feminise boys [36]. These chemicals are now banned in both the USA and European Union [37,38]. Current knowledge of environmental exposure to neurotoxic substances, particularly in infants, is limited because hazardous exposure may not be immediately apparent [39].

### Which part(s) of infant's CNS might be affected by neurotoxic substances, leading to AIS?

At present, we can only speculate about which parts of an infant's CNS might be affected by neurotoxic substances. Although the biological processes that underlie the development of idiopathic scoliosis in adolescents, particularly girls, are poorly understood [40,41], there is increasing evidence and growing support for the possibility of an underlying neurological disorder [40-48]. The evidence relates to the spinal cord [43,44], hind brain [46], motor control [45], motor cortex [49], supplementary motor area [50], and vestibular system [6,51]. The neurogenic hypothesis for AIS outlined here is that neurotoxins from heated indoor swimming pools contribute to dysfunction of the brain and/or spinal cord leading to AIS in some patients.

### Testing the hypothesis

Confirmation of the epidemiological findings clearly needs to be done by other workers. But the absence of such confirmation should not delay testing of the neurogenic hypothesis for AIS. Some research for consideration is outlined below.

### Neurotoxic substances produced in swimming pools

The degree of exposure to neurotoxic substances that is needed to cause sublethal neural tissue damage is unknown and very difficult to quantify. Chloroform concentrations in pools will be up to 100 ppb [14-16], and cyanogens chloride concentrations up to 140 ppb [11-13]. Concentrations in the pool heated pool atmosphere could reach 500-1000 μg/m^3 ^in air. Given that these concentrations are set for adults rather than for infants, research in this area is needed.

### Entry of neurotoxic substances to infant's body and CNS, and the CNS's vulnerability to such assaults with delayed effects

Passage of neurotoxic substances from environment to the infant's body and CNS can each occur through several routes. Without samples of environmental air, alveolar air, and plasma from infants, the potential hazardous effects of THMs on infants is unknown. To the best of knowledge no research has been published to assess any delayed effects of THMs on infants.

### Experimental neurotoxicity testing in animals

Experts testing the neurotoxicity of the pool-derived substances in animals with respect to scoliosis may wish to consider including mice vulnerable to scoliosis to establish whether, and if so, where in the CNS, the neurotoxic substances may become localised.

### Implications of the hypothesis

Chlorination of the water in heated indoor swimming pools is paramount to ensure pool hygiene. THMs are produced as a result of chlorination. This paper addresses the known neurotoxins that could leave the primitive developing structures of the infant's CNS vulnerable as a result of the inability of the barrier mechanisms to protect. The infant's CNS vulnerability is well known, so understanding of its fragility is necessary for future interpretation of any neurotoxic effects. As with all toxic assaults on the immature CNS, it must be defined by both dose and duration. The time lag is addressed, because studies of the toxicity of xenobiotic compounds in children are known to have potential to manifest later in life.

The neurogenic hypothesis involving delayed epigenetic effects of neurotoxic substances can be tested. If confirmed, there is potential to prevent some children from developing AIS. There could be other environmental factors acting in the first year of life to initiate AIS that differ around the world, with one environmental factor detected in Scotland involving heated indoor swimming pools.

## List of abbreviations

AIS: adolescent idiopathic scoliosis; BBB: blood-brain barrier; B-CSF-B: blood-cerebrospinal fluid barrier; CNS: central nervous system; CSF: cerebrospinal fluid; CVOs: circumventricular organs; THMs: trihalomethanes;

## Competing interests

The author declares that they have no competing interests.

## Authors' contributions

MEM was responsible for all aspects of this paper and read and approved the final version.
